# A Narrative Review of the Genetic Architecture of Systemic Hypertension: From Candidate Genes to Biobank-Scale Genome-Wide Association Studies and Sequencing, With Translational Pathways to Precision Care

**DOI:** 10.7759/cureus.99825

**Published:** 2025-12-22

**Authors:** Abdullah Aldhufairi, Dhari Alenezi, Ebrahim Ebrahim, Ali Khalaf, Ameer Alkhabbaz

**Affiliations:** 1 General Practice, Al Jahra Hospital, Al Jahra, KWT; 2 General Practice, Jordan University of Science and Technology, Irbid, JOR

**Keywords:** genetic architecture, gwas, polygenic risk, precision medicine, resistant hypertension, systemic hypertension, wes/wgs

## Abstract

Systemic hypertension arises from the interplay of numerous common and rare genetic variants spanning vascular, renal, endocrine, metabolic, and immune pathways. Modern genomic approaches triangulate evidence from candidate gene studies, biobank-scale genome-wide association studies (GWAS), and whole-exome or whole-genome sequencing, enabling stronger mechanistic inference. In this narrative synthesis, we focused on recent human studies emphasizing candidate gene analyses, GWAS, and sequencing efforts in hypertension, extracting data on study design, populations, key variants, and implicated biological pathways. Across methodologies, genetic evidence consistently supported central roles for endothelial nitric-oxide biology (NOS3) and oxidative or tonic regulation of arteriolar resistance (PRKG1, CYBA, and CYP4A11), alongside contributions from lipid-handling genes (ApoB and PCSK9) and mitochondrial or smooth-muscle regulators (HSG and MFN2). GWAS conducted across diverse ancestries repeatedly mapped blood pressure variation to vascular calcium dynamics (ATP2B1 and CACN* loci), renal tubular transport mechanisms (UMOD and SLC4A7), renin-angiotensin-aldosterone system-related steroidogenesis (CYP17A1 and CYP11B2), and immune remodeling pathways (SH2B3), with several loci demonstrating sex- or ancestry-specific modulation and enrichment in resistant-hypertension cohorts, particularly within calcium-handling and steroidogenic pathways. Sequencing studies further identified rare, functional, and ancestry-specific variants, including large blood pressure-lowering alleles and signals enriched in Middle Eastern populations, that refine biological mechanisms and support population-tailored risk stratification. Overall, convergent evidence across genetic approaches highlights four translationally actionable systems, such as vascular calcium handling, renal salt and bicarbonate transport, adrenal steroidogenesis, and immune or inflammatory tone, supporting the development of ancestry-aware polygenic risk tools, genetic sub-phenotyping (including resistant hypertension), and mechanism-aligned therapeutics as key steps toward precision hypertension care.

## Introduction and background

Systemic hypertension (high blood pressure) is a major global health challenge, affecting about 1.4 billion people worldwide, roughly one in three adults [1]. It has become the leading risk factor for mortality globally, with recent Global Burden of Disease analyses attributing approximately 10 million deaths per year to high blood pressure [2]. This accounts for nearly one-fifth of all deaths and underscores hypertension’s central role in heart disease, stroke, and other related complications [3]. The condition also imposes a tremendous economic burden, as managing hypertension and its consequences consumes around 10% of global health care spending, an estimated $800 billion annually [4]. These updated figures highlight the enormous worldwide impact of hypertension in terms of prevalence, mortality, and cost, emphasizing its significance as a major global public health priority.

Clinical hypertension management faces notable limitations. Conventional approaches rely on fixed blood pressure thresholds (such as 140/90 mmHg) that apply a uniform definition of hypertension, overlooking individual cardiovascular risk differences and population variability [5-7]. For instance, lowering the diagnostic cutoff to 130/80 mmHg, as recommended in the 2017 ACC/AHA guidelines, helped identify more high-risk patients but also raised concerns about potential overdiagnosis and overtreatment among low-risk individuals [5,6]. Standard treatment algorithms similarly lack true personalization; guideline-driven therapy often fails to account for patient-specific factors or heterogeneity in drug response [8,9]. Consequently, many patients struggle to achieve target blood pressure levels, and a substantial subset develops resistant hypertension, defined as uncontrolled BP despite the use of multiple medications. These limitations contribute to suboptimal outcomes, with fewer than half of hypertensive patients attaining guideline-recommended control, resulting in preventable cardiovascular events and organ damage. Recent evidence and expert analyses have highlighted these shortcomings, emphasizing the need for more individualized, risk-based diagnostic and therapeutic strategies to improve hypertension management and patient outcomes.

The aim of this study is to summarize modern genetic evidence on hypertension from different types of research, including studies that focus on specific genes (candidate gene studies), large genome-wide association studies (GWAS), and whole-exome or whole-genome sequencing. By combining these sources, the study highlights key biological pathways that influence blood pressure, such as how blood vessels control calcium, how the kidneys regulate salt, and how hormones and immune signals affect vascular tone. It also explores differences across populations and sexes and discusses how these findings may support future clinical applications, including improved risk prediction and more personalized treatment for resistant hypertension.

## Review

Methodology

Design and Scope

This work is a narrative synthesis of human genetic studies of systemic hypertension as compiled in the manuscript. We included (1) candidate gene studies grounded in vascular, renal, metabolic, and mitochondrial biology; (2) large GWAS (single- and multi-ancestry; discovery, replication, and integrative analyses); and (3) sequencing-based reports (whole-exome/whole-genome sequencing, WES/WGS) that identify rare or ancestry-enriched variants. Articles emphasized in the tables and text were treated as index studies for each pathway.

Eligibility Criteria and Study Selection

**Figure 1:**
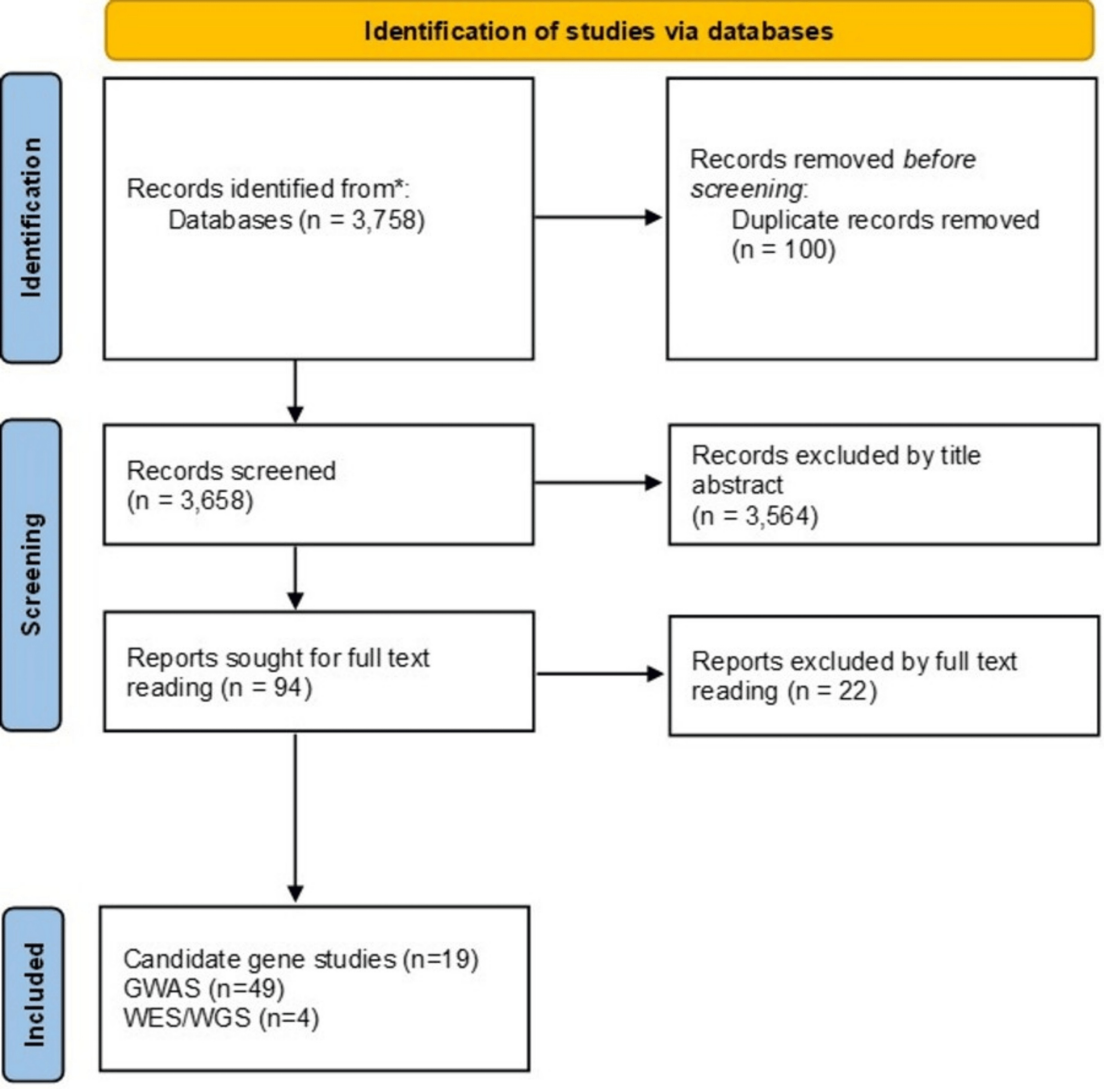
PRISMA 2020 flow diagram for studies included in this study GWAS: genome-wide association studies; PRISMA: Preferred Reporting Items for Systematic reviews and Meta-Analyses; WES: whole exome sequencing; WGS: whole genome sequencing

Inclusion required human participants, explicit blood pressure or hypertension outcomes, and a report of specific loci/variants or gene-based signals with plausible mechanistic mapping. We prioritized recent (last ~10 years) studies for candidate gene and GWAS sections and included key sequencing studies illustrating rare-variant and regional insights. A systematic search was conducted in accordance with the Preferred Reporting Items for Systematic reviews and Meta-Analyses (PRISMA) 2020 guidelines to identify genetic studies on systemic hypertension across three major categories: candidate gene analyses, GWAS, and WES/WGS. The literature search used simple keyword combinations including “hypertension”, “essential hypertension”, “blood pressure”, “genetics”, “candidate gene”, “polymorphism”, “SNP”, “GWAS”, “genome-wide association”, “whole-exome sequencing”, and “whole-genome sequencing”. The search was conducted across three major databases, such as PubMed, Web of Science, and Scopus, using the previous keyword. The search retrieved 581 candidate gene studies, 2,669 GWAS, and 508 WES/WGS articles, which underwent title-abstract screening and full-text assessment based on predefined eligibility criteria. Following this process, 19 candidate gene studies, 49 GWAS, and four WES/WGS studies met inclusion requirements for synthesis. The full study selection pathway, including identification, screening, eligibility, and final inclusion steps, is illustrated in Figure 1.

Data Extraction and Synthesis

From each study, we abstracted population/ancestry, sample size, design, top variant(s)/gene(s), principal findings, and inferred biological pathway(s). For GWAS, we grouped signals into recurrent axes, such as vascular Ca²⁺ handling, renal transport, adrenal/renin-angiotensin-aldosterone system (RAAS), and immune signaling, and noted sex- or ancestry-specific effects when described. For sequencing, we highlighted rare functional alleles and ancestry-enriched signals and mapped them to endothelial, neuronal, ion-channel, and RAAS pathways. Synthesis emphasized cross-method concordance and translational implications (polygenic risk, resistant-hypertension genetics, and mechanism-aligned therapy).

Limitations

As a narrative review of curated studies, this methodology does not perform formal risk-of-bias scoring, meta-analysis, or exhaustive database searches; effect sizes and heterogeneity are presented as reported. Nonetheless, convergence across orthogonal methods (candidate gene → GWAS → WES/WGS) and across ancestries strengthens mechanistic inference.

Candidate gene studies in systemic hypertension

The search for genetic determinants of systemic hypertension has long relied on the candidate gene approach, which investigates biologically plausible genes within known physiological pathways of blood pressure regulation. Before the genome-wide era, this strategy served as a key means to link specific molecular mechanisms, such as endothelial function, vascular tone, and oxidative stress, to the clinical phenotype of hypertension. Although limited by sample size and heterogeneity, candidate gene studies remain valuable for identifying mechanistic insights and population-specific risk alleles. Table 1 summarizes several representative studies across diverse ethnic groups, highlighting the polygenic and context-dependent nature of hypertension susceptibility.

**Table 1:** Candidate gene studies in systemic HTN in the last 10 years (human studies only) ANP: atrial natriuretic peptide; BP: blood pressure; CKD: chronic kidney disease; DBP: diastolic blood pressure; DM: diabetes mellitus; DKD: diabetic kidney disease; ENaC: epithelial sodium channel; HTN: hypertension; LDL: low-density lipoprotein; MAP: mean arterial pressure; SBP: systolic blood pressure; SNP: single nucleotide polymorphism; T2DM: type 2 diabetes mellitus; VNTR: variable number tandem repeat

Gene(s) studied	Population/sample	Design	Polymorphisms/SNPs	Main findings	Conclusion
FGFR2 and FGFR4 [10]	Korean, 58,698 individuals	Case-control	FGFR2 (rs199545667, rs4752570), FGFR4 (rs351855)	FGFR2 (rs199545667, rs4752570) were linked to HTN and osteoporosis, and to height and HTN; FGFR4 (rs351855), a missense variant, was linked to HTN and height.	FGFR2 and FGFR4 variants predispose to HTN, possibly via metabolic effects involving calcium signaling, MAPK, and RAS pathways
ERBB3/PA2G4 [11]	White Southern Brazilians, 976 participants	Case-control	ERBB3 (rs705708 A allele)	The ERBB3 rs705708 A allele was linked to a lower prevalence of arterial HTN and other microvascular complications in patients with type 1 DM.	Protective role of the ERBB3 rs705708 A allele against microvascular complications (retinopathy, nephropathy) and HTN
NOS3 (India) [12]	299 HTN patients vs 294 normotensive controls (India)	Case-control	E298D (rs1799983)	The E298D (rs1799983) T allele was linked to HTN.	eNOS dysfunction contributes to BP elevation
NOS3 (Sudanese) [13]	Sudanese, 157 HTN vs 85 controls	Case-control	rs2070744, rs1799983, intron 4 VNTR	The T-786C C allele was associated with increased HTN risk (OR ~2); other SNPs showed no association	Confirms the role of NOS3 in African populations
PRKG1, CYP4A11, and CYBA [14]	2,057 unrelated northern Han Chinese	Case-control	PRKG1 (rs1904694, rs7897633), CYP4A11 (rs1126742), CYBA (rs4673)	PRKG1 (rs1904694, rs7897633), CYP4A11 (rs1126742), and CYBA (rs4673) were linked to HTN. CYBA (rs4673) was associated with MAP elevation in hypertensives and SBP reduction during diuresis shrinkage, while CYP4A11 (rs1126742) was associated with DBP and MAP reduction in normotensives.	Confirms the importance of multiple candidate genes in salt-sensitive BP, including PRKG1 (vascular smooth muscle relaxation), CYBA (oxidative stress), CYP4A11 (renal sodium handling), and AGTR1 (renin-angiotensin pathway).
ApoB/PCSK9 [15]	Chinese T2DM, 575 DKD vs 653 controls	Case-control	ApoB (rs1042034, rs679899, rs676210, rs1367117, rs12720838); PCSK9 (rs662145, rs45448095, rs11583680)	The ApoB haplotype T-A-G-T (harboring rs1042034 and rs12720838 variants) was significantly associated with HTN.	ApoB variants predispose to DKD and HTN via lipid- and BP-related effects.
HSG/Mfn2 [16]	Chinese, 402 hypertensive vs 267 controls	Case-control	HSG (rs873457, rs2236384, rs4846085, rs1474868)	All four SNPs increased essential HTN risk; effects were male-specific, and the haplotype C-G-T-T-T-G conferred higher risk.	HSG variants influence HTN via vascular smooth muscle proliferation and insulin resistance.
ECE1 [17]	Han Chinese, 398 hypertensive patients vs 596 controls	Case-control	10 tag SNPs (rs212544, rs2076280, rs115071, rs2076283, rs9426748, rs11590928, rs212515, rs2236847, rs2282715, rs2774028)	Sex-specific associations were observed: rs115071 was protective in men, while rs212544 and rs2236847 increased risk in women.	ECE1 polymorphisms are linked to HTN via the endothelin pathway.
NEDD4L [18]	Han Chinese CKD patients (n=623)	Case-control	rs4149601, rs2288774	The rs4149601 AA genotype markedly increased HTN risk, while rs2288774 showed no association.	NEDD4L influences salt-sensitive HTN via ENaC regulation.
HYGEF (multi-gene) [19]	Italian adolescents (n=2638)	Case-control, youth cohort	15 SNPs in 13 genes (ADD1, ADD2, ADD3, LSS, KL, CYP11A1, CYP11B2, HSD3B1, NEDD4L, PRKG1, UMOD, ACE, ABCB1)	The LSS rs2254524 A allele was associated with higher SBP (+1.9 mmHg) and DBP (+1.1 mmHg). HSD3B1 rs6203 and rs2236780 and PRKG1 rs1904694 were associated with SBP; CYP11B2 rs1799998 and NEDD4L rs4149601 with DBP. KL rs9536314 was associated with 24-h urinary sodium excretion and the Na/K ratio. ADD1 rs4961 was associated with the Na/K ratio in males and showed epistatic interaction with HSD3B1 rs10923835.	Genetic risk for HTN is evident in adolescence.
PPARD [20]	Korean adults, 1,793 (434 HTN, 1,359 controls)	Case-control	rs7770619	The CT genotype was protective against HTN and was associated with lower plasma malondialdehyde and fasting glucose and higher adiponectin levels.	The PPARD variant protects against HTN via oxidative stress and metabolic regulation.
ACE [21]	Northern Han Chinese, 2,040 adults	Case-control	A2350G, I/D	The D allele and G allele increased HTN risk, with the G-D haplotype showing the strongest association.	ACE polymorphisms contribute significantly to essential HTN in Chinese populations.
EPHA6 [22]	Korean adults, 2,146 (541 HTN, 1,605 controls)	Case-control	rs4857055	The TT genotype increased HTN risk and was linked to higher apoB, triglycerides, and smaller LDL particle size.	EPHA6 contributes to HTN via vascular remodeling and dyslipidemia.
PAM [23]	Korean adults, 2,153 (543 HTN, 1,610 controls)	Case-control	rs13175330	The G allele and GG genotype increased HTN risk (OR 1.6) and were linked to higher SBP and DBP, insulin resistance, and oxidized LDL.	The PAM polymorphism alters ANP secretion and metabolic traits.
ESR2 (ER-β) [24]	584 European/Middle Eastern and African patients and 662 Mexican American patients	Case-control, dietary intervention	rs10144225 (ESR2)	The G allele increased salt sensitivity in premenopausal women and was linked to aldosterone dysregulation and HTN.	ESR2 variation influences salt-sensitive BP in estrogen-replete women.
CYP3A5 [25]	Ghanaians, 881	Case-control, community sample	rs776746, rs10264272	No significant BP differences were observed by genotype, although a trend toward higher HTN prevalence was noted in non-expressers.	No strong support for a role of CYP3A5 in African HTN.
SLC35F3 [26]	Han Chinese, 2,527	Case-control	rs34032258	G-allele carriers showed increased DBP and altered renal indices, with stronger associations observed in obese individuals.	The SLC35F3 variant contributes to diastolic HTN, possibly via thiamine transport.
GNB3 [27]	Egyptians, 438 (216 HTN, 222 controls)	Case-control	C825T (rs5443)	The T allele increased HTN risk (OR 1.5), with TT homozygotes showing an OR of 2.3; the variant was also linked to higher BMI.	The GNB3 splice variant increases vascular reactivity and obesity-related HTN.
RANK and OPG [28]	695 Spanish women	Case-control	RANK (rs884205), OPG (rs4876869)	RANK rs884205 was linked to HTN, including both SBP and DBP.	RANK rs884205 is a potential candidate SNP influencing blood pressure in women.

Growth Factor and Receptor Pathways

Among the most consistently implicated genes are those regulating vascular growth and remodeling. The fibroblast growth factor receptor genes (FGFR2 and FGFR4) were investigated in a large Korean cohort comprising 58,698 individuals [10]. Variants FGFR2 rs199545667 and rs4752570, along with FGFR4 rs351855, showed significant associations with elevated blood pressure and hypertension prevalence. These findings suggest that fibroblast growth factor signaling contributes to vascular wall integrity and smooth muscle proliferation, thereby influencing peripheral resistance. The scale and homogeneity of the Korean study lend strength to these observations and reinforce the importance of vascular remodeling pathways in systemic blood pressure control.

Similarly, the ERBB3 and PA2G4 genes, both encoding growth-factor-related receptors, were examined in 976 White Southern Brazilians [11]. The ERBB3 rs705708 A allele was associated with a lower prevalence of hypertension, indicating a potentially protective role. ERBB3 participates in epidermal growth factor receptor signaling, which modulates endothelial proliferation and inflammation. The identification of a protective allele underscores the possibility that not all receptor polymorphisms increase disease risk; some may enhance receptor efficiency or down-regulate inflammatory cascades, offering resilience to hypertension development. Ethnic-specific effects such as these illustrate how population structure and gene-environment interactions can shape the genetic architecture of hypertension.

Endothelial Nitric Oxide Synthase (NOS3) Variants

Endothelial dysfunction is another well-recognized mechanism underlying elevated blood pressure, and several studies have confirmed the contribution of NOS3 gene variants across different ethnicities. In an Indian cohort of nearly 600 participants, the E298D (rs1799983, T allele) was significantly associated with hypertension, suggesting impaired nitric oxide (NO) bioavailability [12]. The same gene was explored in a Sudanese population (157 hypertensive vs 85 control subjects), where the T-786C C allele increased hypertension risk with an OR of approximately 2.0 [13]. Additional intronic and VNTR polymorphisms showed variable associations. These results collectively affirm the pivotal role of endothelial NO production in vascular homeostasis and demonstrate consistent replication of NOS3 effects across populations, albeit with differing magnitudes. Importantly, ethnic variation in linkage disequilibrium (LD) patterns and environmental exposures, such as salt intake or physical activity, may modify the penetrance of NOS3 variants, explaining discrepancies between studies.

Signal Transduction and Oxidative Stress Genes

The complexity of hypertension pathogenesis is further reflected in multigene studies targeting interrelated pathways. A large case-control study of northern Han Chinese (n = 2,057) simultaneously evaluated PRKG1, CYP4A11, and CYBA genes encoding proteins that regulate vascular smooth muscle contraction, eicosanoid metabolism, and reactive oxygen species generation. Several variants, including PRKG1 rs1904694, CYP4A11 rs1126742, and CYBA rs4673, demonstrated associations with increased hypertension susceptibility [14]. Together, they reinforce the concept that disturbances in vascular tone regulation and oxidative stress act synergistically to elevate systemic resistance. Notably, the involvement of both signaling and metabolic genes suggests that hypertension may arise from coordinated dysregulation of multiple interdependent molecular systems rather than from isolated single-gene defects.

Lipid Metabolism and Secondary Hypertension Mechanisms

Although hypertension is traditionally viewed as a vascular disorder, evidence also implicates lipid-handling genes in its etiology, particularly when comorbid with metabolic disease. In a Chinese cohort of 1,228 participants with type 2 diabetes mellitus, polymorphisms in ApoB and PCSK9 were associated with hypertension and diabetic kidney disease (DKD) [15]. The ApoB haplotype T-A-G-T, harboring variants rs1042034 and rs1367117, conferred a higher risk of DKD and elevated blood pressure. Because both ApoB and PCSK9 regulate low-density lipoprotein metabolism, their variants may promote vascular dysfunction through dyslipidemia-mediated endothelial injury. This observation broadens the pathophysiological spectrum of hypertension to include metabolic and renal mechanisms and highlights the shared genetic architecture between cardiovascular and metabolic diseases.

Mitochondrial and Smooth Muscle Function Genes

A more recent Chinese study identified polymorphisms in HSG (also known as Mfn2), a gene involved in mitochondrial fusion and vascular smooth muscle cell homeostasis, as risk factors for essential hypertension [16]. Four SNPs (rs873457, rs2236384, rs4846085, and rs1474868) increased hypertension risk, with effects most pronounced in male subjects. This finding underscores the emerging recognition of mitochondrial bioenergetics and sex-specific genetic modulation in blood pressure regulation. Mitochondrial dysfunction can impair energy-dependent ion transport and smooth muscle relaxation, providing a plausible mechanistic link between HSG variants and vascular contractility.

Cross-Study Synthesis and Emerging Themes

Despite differences in design and sample size, the studies collectively reveal several consistent themes. First, nearly all implicated genes converge on three core biological pathways, such as (1) endothelial NO signaling [12,13]; (2) vascular remodeling and extracellular matrix turnover [10,11]; and (3) oxidative and metabolic stress regulation [14,15]. This convergence provides functional validation that candidate gene selection based on known physiology remains biologically sound.

Second, the magnitude and direction of genetic effects vary substantially across ethnic groups, highlighting the influence of population-specific allelic distributions and local environmental modifiers. For example, NOS3 variants appear consistently deleterious in both Indian and African populations [12,13], whereas ERBB3 polymorphisms exert protective effects in Brazilians [11]. Such heterogeneity reinforces the need for replication in diverse cohorts and for meta-analytic integration to determine generalizable risk loci.

Third, the results emphasize the polygenic nature of hypertension. No single locus accounts for a large proportion of heritability; instead, each variant exerts a modest effect that becomes clinically relevant only in combination with other genetic or environmental factors. This paradigm aligns with modern GWAS evidence and supports the development of polygenic risk scores that integrate multiple candidate gene signals into a composite genetic risk estimate.

Finally, the translational potential of these findings remains limited but promising. As genotyping costs fall, clinically validated variants, particularly those in NOS3, MMPs, and CYP4A11, may contribute to personalized hypertension management, guiding early screening and pharmacogenetic interventions. However, larger multiethnic cohorts, functional validation, and longitudinal follow-up are required before clinical application.

GWAS of systemic hypertension

All GWAS investigating systemic hypertension are presented in Table 2.

**Table 2:** GWAS studies in systemic HTN in the last 10 years (human studies only) aTRH: apparent treatment-resistant hypertension; BBJ: BioBank Japan; BCAMS: Beijing Child and Adolescent Metabolic Syndrome study; BP: blood pressure; CHARGE: Cohorts for Heart and Aging Research in Genomic Epidemiology; COGENT: Continental Origins and Genetic Epidemiology Network; DBP: diastolic blood pressure; DT: distal tubule of the kidney; EAGLE: Early Genetics and Lifecourse Epidemiology Consortium; FINRISK: Finnish Risk Factor Study; GERA: Genetic Epidemiology Research on Adult Health and Aging; GRS: genetic risk score; GWAS: genome-wide association study; HTN: hypertension; KoGES: Korean Genome and Epidemiology Study; MAP: mean arterial pressure; MR: mendelian randomization; NHGRI-EBI: National Human Genome Research Institute: European Bioinformatics Institute; NO: nitric oxide; PRS: polygenic risk scores; RAAS: renin-angiotensin-aldosterone system; SBP: systolic blood pressure; SNP: single-nucleotide polymorphism; TAL: thick ascending limb of the loop of Henle; UKB: UK Biobank

Study ID	Population/sample	Design	Polymorphisms/SNPs/genes	Main findings	Main pathway involved	Conclusion
Lidani et al. (2025) [29]	Multi-Ethnic Study of Atherosclerosis; n = 4,831 participants (White, Black, Hispanic, and Chinese)	Observational cross-sectional population-based GWAS	rs4762 (G>A) in AGT exon; rs5050 (T>G) in AGT promoter; rs16852311 (G>C)	Both rs4762 and rs5050 influenced BP and HTN indirectly via plasma AGT levels, rather than directly	Adrenal steroidogenesis/RAAS (aldosterone/angiotensin)	Variants in AGT influence circulating angiotensinogen, mediating effects on BP and HTN risk
Li et al. (2024) [30]	Hypertension GWAS: UK Biobank European participants; Epigenetic-aging GWAS: meta-analysis of 28 European cohorts	GWAS	rs1849209 (LINC01478), rs1982200 (DNAJC27-AS1), rs2859868 (MECOM), rs7223364 (LSMD1), rs12940887 (ZNF652), rs6456686 (SCGN), rs11944870 (COPS4), rs17126990 (SLC35F1), rs2872818 (GPR126), rs1888925 (ADGRG6), rs11190870 (LRP1B), rs11227161 (CACNB2), rs77846625 (CYP1B1), rs2472297 (CYP1A1/CYP1A2 cluster), rs72800365 (TBL1XR1), rs12301816 (APBB2), rs6765567 (KCNK9), rs74409485 (OR5K1), rs11582590 (OR52B6), rs16841150 (CYP2U1), rs1759340 (DAGLA), rs61881106 (FBXL17), rs2475795 (TMEM108), rs72698845 (CHRNA9), rs16852311 (ANKRD36B)	Identified 32 genomic loci jointly associated with HTN and epigenetic aging; 25 were novel for HTN. Nine of 32 loci validated in both the SBP and DBP datasets	Vascular calcium handling/smooth-muscle contractility	The study confirms HTN’s high polygenicity (~2,800 causal variants) and reveals shared genetic architecture with epigenetic aging. Twenty-five novel HTN loci were identified, several involving sensory-perception and neurovascular pathways
Pozarickij et al. (2024) [31]	100,453 adults from the China Kadoorie Biobank	Primary GWAS with follow-up MR analyses	rs11759263 (FIGNL1), rs4913045 (PIK3C2G), rs10460736 (STK38L), rs1044041 (SLC35F3), rs72765725 (COL21A1), rs956913 (ARHGEF12), rs11161729 (TMEM182), rs72732141 (WNT2B), rs13364845 (SLC16A9), rs3827647 (LMX1B), rs17579169 (GPRC5C), rs2862353 (SSPN), rs2018421 (SOX17)	Identified 128 independent loci associated with BP traits; 74 newly reported associations, and 13 genomic regions not previously linked to any BP trait in any ancestry	Unspecified/association not clearly mapped	The findings demonstrated stronger heritability and larger variant effect sizes in Chinese participants compared to European and Japanese cohorts
Tsare et al. (2024) [32]	70 previously published GWAS studies on blood pressure (2007-2023) across 14 global populations (over 1.5 million participants)	Large-scale integrative analysis combining 70 GWAS studies on blood pressure (2007-2023) across 14 global populations	Most frequently replicated SNPs: rs17249754 (ATP2B1), rs11191548 (CNNM2), rs3184504 (SH2B3), rs1458038 (intergenic), rs880315 (CASZ1), rs13107325 (SLC39A8), rs167479 (RGL3), rs16998073 (intergenic)	6,687 genome-wide significant SNPs; 1,167 protein-coding genes; top genes by SNP density: ULK4 (276), ZNF831 (124), FTO (103), SLC4A7 (85), MSRA (82), CLCN6 (79), PINX1 (68), CNNM2 (59), CABCOCO1 (58); strongest associations: ATXN2 and SH2B3	Vascular calcium handling/smooth-muscle contractility; renal tubular Na/Cl/HCO3 transport (TAL/DT); immune/inflammatory signaling (e.g., SH2B3); cerebrovascular/endothelial remodeling	HTN is highly polygenic, with variants distributed genome-wide and converging on ion transport, vascular signaling, and metabolic pathways. Integration of GWAS with eQTL and PPI data revealed that genes such as ATP2B1, CNNM2, and SH2B3 are not only statistically associated but also functionally interconnected regulators of vascular tone
Singh et al. (2023) [33]	10,775 adults from sub-Saharan Africa (Kenya, Ghana, Burkina Faso, South Africa); meta-analyzed with UK Biobank African ancestry (n = 3,058) and Uganda Genome Resource (n = 6,400)	Primary GWAS meta-analysis of HTN and BP traits	Novel genome-wide significant SNPs: rs77846204 (near P2RY1), rs115808349 (near LINC01256/ELL2P2)	rs77846204 (near P2RY1) associated with systolic BP, also with DBP and MAP; rs115808349 (near LINC01256/ELL2P2) associated with pulse pressure and resistance to antihypertensive medication	Unspecified/association not clearly mapped	Identified two novel BP-associated loci unique to African populations; highlighted low transferability of PRS from non-African ancestries and the need for large African GWAS to uncover ancestry-specific HTN biology
Yamazaki et al. (2023) [34]	32,239 Japanese participants in the BioBank Japan cohort	GWAS focused on pharmacogenomic response in HTN	rs6445583 (CACNA1D); rs12308051 (intergenic, chr12); rs35497065 (FOXA3); rs11066280 (HECTD4)	rs6445583 (CACNA1D) - ARB resistance; rs12308051 (intergenic, chr12) - ARB resistance; rs35497065 (FOXA3) - CCB resistance; rs11066280 (HECTD4) - αβ-blocker resistance	Vascular calcium handling/smooth-muscle contractility; growth-factor/ubiquitin signaling (FGF5, HECTD4)	Resistant HTN appears to result from the combined burden of BP susceptibility alleles and alleles influencing reduced drug efficacy
Ivanova et al. (2023) [35]	1,405 unrelated Caucasian participants (939 hypertensive, 466 controls) from Central Russia	Case-control replication GWAS-based study	Significant or interacting SNPs: rs1173771 (AC026703.1), rs1799945 (HFE), rs805303 (BAG6), rs932764 (PLCE1), rs4387287 (OBFC1), rs7302981 (CERS5), rs167479 (RGL3)	rs1799945 (C/G) - HFE gene: GG genotype significantly associated with increased arterial HTN risk. The strongest synergistic pair linked to arterial HTN was rs805303 (BAG6) × rs7302981 (CERS5)	Unspecified/association not clearly mapped	The study confirms the HFE rs1799945 (C/G) polymorphism as an independent risk factor for arterial HTN and underscores the interactive polygenic and immunogenetic mechanisms underlying AH in the Russian Caucasian population
Naito et al. (2023) [36]	Japanese (BBJ + Hiroshima University), UK Biobank, and FinnGen (total = 816 PA cases + 425,239 controls)	Cross-ancestry GWAS meta-analysis	rs3790604 (WNT2B 1p13); rs2023843 (HOTTIP 7p15); rs4980379 (LSP1 11p15); rs35486 (TBX3 12q24); rs35442752 (RXFP2 13q12); rs145725189 (CYP11B1/CYP11B2 8q24); rs78785501 (DPP10 2q14)	Identified variants were linked to PA. rs78785501 (DPP10 2q14) linked to BAH	Adrenal steroidogenesis/RAAS (aldosterone/angiotensin)	Demonstrated genome-wide germline risk architecture of PA, distinct from yet contributory to HTN. Highlighted WNT/β-catenin pathway and CYP11B2-related steroidogenic regulation as causal axes. A large subset of BP GWAS signals may act through genetic susceptibility to PA
Udosen et al. (2023) [37]	80,950 individuals of African ancestry from six cohorts: APCDR (Uganda, South Africa, Nigeria, Ghana, Kenya), UK Biobank (African subset), and Million Veteran Program (MVP)	GWAS - meta-analysis and multivariate GWAS	rs77534700 (AC074290.1); rs562545 (MOBP); rs138493856 (DNAJC17P1/GLULP6); rs139235642 (RRM2); rs72619992 (LOC105377644)	These novel genetic variants were associated with SBP and DBP	Unspecified/association not clearly mapped	Provides the largest African-ancestry GWAS of BP to date, identifying five novel variants. Integration of multivariate GWAS enhances discovery power and emphasizes the importance of ancestral diversity in global HTN genetics
Zhou et al. (2023) [38]	NHGRI-EBI GWAS Catalog (SNPs for obesity and HTN), supported by animal models (spontaneously hypertensive rats and ob/ob mice)	Integrative GWAS and expression analysis (bioinformatic + experimental validation)	rs2930176 (RFT1); rs11775334 (AF131215.9); rs2116830 (KCNMA1 locus); rs3802230/rs1802127 (CACNA1D); rs2186471 (MPDZ); rs9922316 (WWOX)	rs2930176 (RFT1) and rs11775334 (AF131215.9) were novel variants linked to HTN	Vascular calcium handling/smooth-muscle contractility	Identified five overlapping genes (KCNMA1, MPDZ, TCF21, WWOX, CACNA1D) forming the shared genomic background of obesity-related HTN. Reduced renal KCNMA1 expression may serve as a molecular marker and therapeutic target for hypertensive obesity
Xiao et al. (2022) [39]	Chinese Han adults (n = 586 aTRH cases, 871 controls) for discovery; validation cohort = 65 aTRH, 96 hypertensive, 100 controls	Human GWAS with eQTL and expression validation	SDC3, LAPTM5: rs7542771, rs10798802, rs11299707, rs878465, rs10798803-rs10798805 (14 linked SNPs); UGT2A1/UGT2B4: rs1432330, rs4148279, rs4148277, rs7672805, rs10000435, rs1432332; FTMT: rs1876648, rs10056108; NIPA1: rs7181789	Total significant SNPs: 23 (3 genotyped, 20 imputed) across four loci. Lead locus on 1p35 contained variants (rs7542771 and linked SNPs) that reduced SDC3 expression and increased LAPTM5 expression	Unspecified/association not clearly mapped	Novel genetic association between SDC3 and treatment-resistant HTN
Jia et al. (2022) [40]	9,370 participants from the UK Biobank, all European (White British) ancestry with ≥3 blood pressure readings across four follow-ups (2006-2020)	Human GWAS	rs574087 (CCDC88B), rs1229536170 (BAD), rs11542299 (GPR137), rs12146487 (PLCB3), rs574835 (RPS6KA4), rs11941467 (WWC2)	SBPV: rs574087 (CCDC88B), rs1229536170 (BAD), rs11542299 (GPR137), rs12146487 (PLCB3), rs574835 (RPS6KA4); DBPV: rs11941467 (WWC2)	Immune/inflammatory signaling (e.g., SH2B3)	First GWAS of BP variability identified novel loci including CCDC88B, RPS6KA4, and WWC2, suggesting inflammation, apoptosis, and vascular signaling play central roles in BP fluctuation. Provides new targets for understanding HTN-related cardiovascular instability
Lee et al. (2022) [41]	5,211 normotensive Korean adults (KoGES cohort)	Population-based prospective cohort GWAS	rs11726091 (MSX1), rs8137145 (SGSM1), rs17038966 (intergenic, chr4q25), rs145286444 (TTL1), rs2118663 (FAM114A2/MFAP3), rs12336898 (SPTAN1), rs1938859 (TRPC6), rs7968218 (CRADD), rs117246401 (ZNF271P/INO80C)	Nine SNPs associated with incident HTN, with rs12336898 in SPTAN1 showing the strongest association	Adrenal steroidogenesis/RAAS (aldosterone/angiotensin)	Identified novel loci associated with renin-stratified HTN, particularly implicating SPTAN1 (rs12336898) as a key gene for low-renin HTN
Cho et al. (2021) [42]	13,926 Korean individuals (6,402 males; 7,524 females) from KoGES	GWAS	rs11066015 (ACAD10), rs2074356 (HECTD4), rs11066280 (HECTD4), rs1392550 (RBM46/NPY2R), rs142469845 (INO80), rs16998073 (PRDM8/FGF5)	Male-specific: rs11066015 (ACAD10), rs11066280 (HECTD4), rs1392550 (RBM46/NPY2R). Female-specific: rs16998073 (PRDM8/FGF5), rs142469845 (INO80)	Growth-factor/ubiquitin signaling (FGF5, HECTD4)	Genetic susceptibility to HTN in Koreans is sex-specific, with distinct loci influencing blood pressure regulation differently in men and women
Takahashi et al. (2021) [43]	2,705 resistant HTN cases and 21,296 mild HTN controls - all Japanese participants from BioBank Japan	Case-control GWAS adjusted for age and sex; genotyped using Illumina HumanOmniExpressExome BeadChip; imputed with 1000 Genomes Phase 3	Genome-wide significant: rs1442386 (DLGAP1); Suggestive loci: rs62525059 (CYP11B2), rs3774427 (CACNA1D), rs77163128 (IGFBP3), rs4247284 (ESRP1), rs73324844 (BCAS3), rs2212606 (ERG), rs78813487 (GAB4), rs3732103 (PQLC3), rs200741614 (LOC105369874), rs11619475 (MED4)	rs1442386 (DLGAP1, chr18p11.3) linked to resistant HTN	Vascular calcium handling/smooth-muscle contractility; adrenal steroidogenesis/RAAS (aldosterone/angiotensin)	Identified a novel locus, rs1442386 in DLGAP1, significantly associated with resistant HTN in Japanese individuals, highlighting a potential neural pathway component in drug-resistant blood pressure elevation
Kolifarhood et al. (2021) [44]	7,694 Iranian adults (≥18 years) from the Tehran Cardiometabolic Genetic Study (TCGS) cohort	Discovery cohort (n = 4,657 for quantitative traits; n = 4,214 for HTN) with replication in 1,618 family-based participants (210 families)	ABHD17C (rs1078107), FBN1 (rs2303505, rs363830, rs363838), ZBED9 (rs450630, rs9501180, rs380914, rs6456825, rs9885928)	rs1078107 (ABHD17C) linked with DBP; rs2303505, rs363830, rs363838 (FBN1) linked with SBP; rs450630, rs9501180, rs380914, rs6456825, rs9885928 (ZBED9) linked with HTN	Unspecified/association not clearly mapped	Three loci-ZBED9, FBN1, and ABHD17C-were associated with BP traits in Iranians, with ZBED9 emerging as a novel HTN gene validated through family-based linkage and cross-ethnic replication
Tan et al. (2021) [45]	Chinese Han population - total 8,751 participants (Discovery: 353 cases/332 controls; Replication: 1,592 cases/1,302 controls; Validation: 3,274 cases/2,734 controls)	Three-stage GWAS for HTN: Discovery (GeneID-I), Replication (NHAPC), Validation (GeneID-II); adjusted for age, age², sex, and top three principal components	rs2064453 (GGT7), rs10847208 (LINC00944), rs28587458 (FSTL5)	Two novel loci-GGT7 (rs2064453) and LINC00944 (rs10847208)-identified for HTN	Endothelial/vascular signaling (NO-cGMP, Src/CSK)	Functional assays suggest the GGT7 risk allele enhances gene expression, downregulates ERK1/2 signaling, and reduces PPP6C levels, contributing to endothelial dysfunction and elevated BP
Li et al. (2021) [46]	380 Chinese twin pairs (243 monozygotic, 137 dizygotic) from the Qingdao Twin Registry, mean age 51.5 ± 7.6 years	Bivariate Cholesky twin model to estimate heritability and genetic correlations for BMI-SBP, BMI-DBP, and SBP-DBP; GWAS conducted in dizygotic pairs with imputation and eQTL analysis	TMEM196 (rs540063109), TENM4 (rs61118809, rs5792827, rs5013388, rs79840843, rs57611205, rs673456, rs12419933), GNGT2 (rs4794029, rs4400367, rs10113750, rs11776003, rs3739327, rs55978930)	TMEM196 (rs540063109) significantly associated with combined BMI-SBP trait; TENM4 variants strongly associated with SBP-DBP correlation	Cardiometabolic cross-talk (adiposity/lipids/glycemia → BP)	Twin-based bivariate GWAS identified TMEM196, TENM4, and functional variants in GNGT2 and SDCBP as shared genetic determinants linking obesity and HTN in the northern Chinese population
Sun et al. (2021) [47]	Up to 128,894 adults from five ancestry groups (African, Asian, European, Hispanic, and Brazilian) across 51 cohorts within the CHARGE consortium	Two-stage meta-analysis: Stage 1: up to 68,450 individuals (31 cohorts); Stage 2: 61,046 individuals (20 cohorts); included both main effects and SNP × psychosocial interaction effects (1-df & 2-df models)	rs77010007 (CSF3R), rs111333873 (PLCL2), rs138187213 (FSTL5), rs9342214 (CASP8AP2), rs201673188 (ACA59), rs140203359 (LIN7A/ACSS3), rs142313940 (SNORD38), rs202048896 (7SK), rs73321585 (CHODL)	Nine new genetic loci associated with BP traits after accounting for gene-psychosocial interactions	Immune/inflammatory signaling (e.g., SH2B3)	Multi-ancestry GWAS identified nine novel BP loci (e.g., PLCL2, FSTL5, LIN7A, CHODL) whose effects were modulated by psychosocial factors, highlighting genetic, neuronal, immune, and stress pathways influencing HTN risk
Irvin et al. (2019) [48]	15 cohorts (10 European ancestry, 5 African ancestry) from the CHARGE consortium	Case-control GWAS meta-analysis; logistic regression adjusted for age, sex, study site, and ancestry components	CASZ1 (rs12046278, rs34071855, rs17035646, rs880315), DNMT3A/DTNB (rs11674660), MYO5B (rs76967376)	All identified genetic loci were linked to aTRH	Cerebrovascular/endothelial remodeling	Multi-ethnic GWAS identified CASZ1 as a robust locus for treatment-resistant HTN and highlighted MYO5B and DNMT3A as additional candidate genes warranting further investigation
El Rouby et al. (2019) [49]	1,194 hypertensive participants (White and Hispanic) with coronary artery disease from INVEST-GENES (discovery) and 585 hypertensive participants from SPS3-GENES (replication); secondary validation in eMERGE cohort (n = 1,946 cases, 471 controls)	Discovery GWAS in INVEST (White and Hispanic subgroups), replication in SPS3, validation in eMERGE; logistic regression adjusted for age, sex, BMI, diabetes, and principal components of ancestry	rs11749255 (MSX2), rs6487504 (IFLTD1), rs324498 (PTPRD), rs16934621 (BNC2)	rs11749255 (MSX2), rs6487504 (IFLTD1), and rs324498 (PTPRD) were significantly associated with resistant HTN and replicated across cohorts	Cerebrovascular/endothelial remodeling	GWAS identified MSX2, IFLTD1, and PTPRD as replicated loci for resistant HTN, supporting a genetic basis for drug-resistant BP via vascular remodeling and signal transduction pathways
Rimpelä et al. (2018) [50]	Finnish hypertensive adults from the GENRES cohort (n = 204), with replication in DYNAMIC (n = 183) and DILGOM (n = 180) studies	GWAS using 24-hour ABPM data collected during placebo periods; replication in independent Finnish cohorts with similar measurements	rs4905794 (BCL11B), rs2119704 (GPR65), rs10817396 (SNX30), rs16984571 (KCNS3), rs12509878 (lncRNA region), rs1230361 (ERAP2)	rs4905794 near BCL11B reached genome-wide significance for SBP dipping and DBP dipping	Unspecified/association not clearly mapped	GWAS identified rs4905794 near BCL11B as a genome-wide significant variant influencing nocturnal BP dipping and left ventricular hypertrophy, highlighting the role of circadian and cardiovascular regulatory genes in HTN pathophysiology
Li et al. (2018) [51]	428 hypertensive and 638 normotensive Mongolian adults from Kerqinzuoyihou Banner, Tongliao, Inner Mongolia, China	Case-control genotyping study; HTN defined as SBP ≥140 mmHg and/or DBP ≥90 mmHg or previous diagnosis; controls SBP/DBP <130/80 mmHg; analysis adjusted for sex and BMI	21 SNPs previously linked to HTN in Tibetans (e.g., rs10507454, rs17010027, rs9407874, rs4547758, rs17136827, rs2045590)	rs10507454 (KCNK3) associated with high diastolic BP, particularly in males; rs17010027 associated with high systolic BP in females; rs4547758, rs17045859, and rs2045590 showed significant genotype differences in male subgroup analyses	Unspecified/association not clearly mapped	This replication GWAS identified sex-specific HTN-associated SNPs (rs10507454, rs17010027, rs4547758) in Mongolians, emphasizing genetic heterogeneity and population-specific BP susceptibility loci
Salo et al. (2017) [52]	6,296 Finnish adults from the FINRISK and Health 2000 population cohorts, replication and validation, totaling >27,000 participants	GWAS of midregional pro-atrial natriuretic peptide (MR-proANP), BNP, NT-proBNP, and BNP:NT-proBNP ratio; followed by replication and BP association testing	NPPA (ANP gene): rs3753584, rs4845875, rs6540997; NPPB (BNP gene): rs198379; PPP3CC (calcineurin subunit gene): rs7000551; GALNT4 (O-glycosylation enzyme): rs11105298, rs61378614	Significant variants included rs3753584, rs4845875, and rs6540997 near NPPA associated with increased ANP (MR-proANP); rs198379 near NPPB linked to BNP; rs7000551 near PPP3CC associated with BNP:NT-proBNP ratio; and rs11105298/rs61378614 near GALNT4 also influencing this ratio. Only NPPA-related alleles that elevate ANP were significantly associated with lower BP and reduced HTN risk	Cardiometabolic cross-talk (adiposity/lipids/glycemia → BP)	Genetic variants increasing ANP (NPPA), but not BNP (NPPB), are associated with lower BP and reduced HTN risk, highlighting ANP as the primary natriuretic peptide influencing BP regulation in humans
Ji et al. (2017) [53]	Data from 43 GWASs of HTN and 18 GWASs of stroke obtained from the NHGRI-EBI GWAS Catalog, covering global populations (European, Asian, African ancestry)	Comparative analysis of GWAS SNPs (p < 1×10⁻⁵) for HTN and stroke; imputation via 1000 Genomes Project (LD ≥ 0.8) using HaploReg 4.1; functional enrichment by DAVID (v6.8) for GO and KEGG analysis	rs3184504 (SH2B3)	rs3184504 (SH2B3) as the principal shared SNP between HTN and stroke. Following imputation, 16 shared genes were detected, including ALDH1A2, ALDH2, APOA5, ATXN2, CNNM2, CUBN, CYP17A1, LPL, NT5C2, PHACTR1, PITX2, SH2B3, and ZPR1	Adrenal steroidogenesis/RAAS (aldosterone/angiotensin), immune/inflammatory signaling (e.g., SH2B3), cardiometabolic cross-talk (adiposity/lipids/glycemia → BP)	SH2B3 and 15 lipid metabolism-related genes are shared determinants of HTN and stroke, highlighting a common genetic pathway involving lipid and vascular regulation
Sofer et al. (2017) [54]	12,278 Hispanic/Latino adults from the U.S. (Mainland: Mexican, Central and South American; Caribbean: Cuban, Dominican and Puerto Rican)	GWAS for 5 BP traits (SBP, DBP, MAP, PP, and HTN status), with stratified analysis by Hispanic subgroup (Mainland vs. Caribbean) and replication in multiple global cohorts	rs190705571 (SCGN, chr6p25.7), NRG3 indel (chr10q23.1), rs73156692 (SLC5A8, chr12), rs1458038 (FGF5, chr4), rs117386367 (chr17), rs11466481 (TGFBR2), rs78701042 (NGF), rs113204208 (intergenic, chr1)	BP-associated SNPs: rs190705571 (SCGN), rs1458038 (FGF5), rs73156692 (SLC5A8), and an intronic variant in NRG3, along with Caribbean-specific loci rs78701042 (NGF) and rs11466481 (TGFBR2)	Growth-factor/ubiquitin signaling (FGF5, HECTD4), cerebrovascular/endothelial remodeling	These genes are tied to renal sodium-phosphate regulation (SCGN/SLC17A family), vascular remodeling (FGF5), neurogenic BP control (NGF), and TGF-β-mediated vascular signaling (TGFBR2), collectively suggesting renal and vascular pathways underlying HTN in admixed Hispanic populations
Wain et al. (2017) [55]	200,000+ participants of European ancestry from the International Consortium for Blood Pressure (ICBP), integrating 1.8 million SNPs from prior GWAS datasets	Genome-wide meta-analysis of previously published BP-related GWAS (SBP, DBP, MAP, PP), aggregating SNP signals into gene-level p-values (n = 17,248 genes tested)	Novel loci: SLC4A7, TBX2, PTK2B, UBE2E2, RAMP1, MECOM, PRKCA, MAPK1, ACVR1C. Previously known loci confirmed: ATP2B1, CACNB2, CYP17A1, SH2B3, CSK, MOV10	Novel HTN-associated genes including SLC4A7, TBX2, PTK2B, UBE2E2, RAMP1, MECOM, PRKCA, MAPK1, ACVR1C, alongside replication of known loci ATP2B1, CACNB2, CYP17A1, SH2B3, CSK, MOV10	Vascular calcium handling/smooth-muscle contractility, renal tubular Na/Cl/HCO3⁻ transport (TAL/DT), adrenal steroidogenesis/RAAS (aldosterone/angiotensin), endothelial/vascular signaling (NO-cGMP, Src/CSK), immune/inflammatory signaling (e.g., SH2B3)	These genes regulate vascular tone, ion exchange, calcium signaling, and endothelial remodeling, suggesting a coordinated network of vascular smooth muscle and cardiac pathways in HTN pathogenesis
Fowdar et al. (2017) [56]	409 hypertensive and 409 normotensive Caucasian Australians (case-control design)	GWAS	rs34870220 (ASGR1), rs4836667 (PRRX2), rs1928277 (NHSL1), rs1599961 (NFKB1), rs11170043 (KRT7), rs12711538 (GLI2), rs11177752 (LRRC10), rs7574068 (LOC100420968), rs1437897 (NCKAP5), rs6576745 (WDR63)	Novel associations: ASGR1, NHSL1, GLI2, LRRC10, NFKB1, ODZ2, GAB2. Previously reported HTN genes confirmed: CDH13, ACE, AGT, ATP2B1, CACNB2, ADRB1, NEDD4, MSRA. No SNPs reached full Bonferroni-corrected genome-wide significance	Vascular calcium handling/smooth-muscle contractility	This GWAS using pooled DNA identified NFKB1 and GLI2 as potential susceptibility genes for essential HTN in Australians. Pooled GWAS effectively estimates allele frequencies and highlights NFKB1 as a promising locus for further research
Warren et al. (2017) [57]	140,886 UK Biobank participants of European ancestry (discovery cohort)	Large-scale GWAS and ExWAS	NOX4, ADAMTS7, SF3A3, GTF2B, METTL21A, PAX2, PDE5A, CACNA2D2, SLC14A2, ACE, ADRA2B, NADK-CPSF3L, THBS2, CFDP1, FBN2, HAND2, GATA2, FN1, EPAS1, INHBA	107 validated SNPs influencing BP, including rs62012628 (ADAMTS7), rs4360494 (SF3A3), rs2289125 (NOX4), rs66887589 (PDE5A), rs743757 (CACNA2D2), rs4308 (ACE), rs2579519 (ADRA2B), rs7236548 (SLC14A2)	Unspecified/association not clearly mapped	This large GWAS identified 107 BP loci (32 novel), notably NOX4, ADAMTS7, and PDE5A, revealing vascular and signaling pathways underlying HTN and potential therapeutic targets for precision cardiovascular medicine
Hoffmann et al. (2017) [58]	99,785 participants in the GERA cohort: 81% non-Hispanic whites, 8% Latinos, 7% East Asians, 3% African Americans, 1% South Asians	GWAS using longitudinal EHR data	rs1322640, rs2104574, rs139491786, rs12556071, rs7632505, rs3184504 (SH2B3), rs11191548 (CYP17A1), rs2681472 (ATP2B1)	75 genome-wide significant loci in GERA: 36 known, 39 novel. Meta-analysis (GERA + ICBP): 36 additional loci (22 replicated in UKB). Combined analysis (GERA + ICBP + UKB): 241 more loci (no replication possible). Total novel loci: 316	Vascular calcium handling/smooth-muscle contractility, adrenal steroidogenesis/RAAS (aldosterone/angiotensin), immune/inflammatory signaling (e.g., SH2B3)	This EHR-based GWAS of >320,000 individuals identified over 300 novel BP loci with strong arterial eQTL enrichment, demonstrating the power of longitudinal EHR data to uncover the genetic architecture of HTN
Parmar et al. (2016) [59]	11,899 children and adolescents from multiple European cohorts participating in the EAGLE Consortium	GWAS performed separately in three age groups (Prepubertal: 4-7 years; Pubertal: 8-12 years; Postpubertal: 13-18 years) and meta-analyzed	rs1563894 (ITGA11), rs872256 (near SMARCA/VLDLR)	Two genome-wide significant variants - rs1563894 (ITGA11) and rs872256 (near SMARCA/VLDLR) - influencing systolic BP in prepubertal and pubertal children, respectively	Cardiometabolic cross-talk (adiposity/lipids/glycemia → BP)	ITGA11 and SMARCA/VLDLR are novel loci influencing childhood systolic BP, showing that genetic regulation of HTN begins early and evolves with development
Diver et al. (2016) [60]	Normotensive discovery group: 60 healthy Caucasian volunteers (West of Scotland); hypertensive validation group: 232 individuals from the BRIGHT study. European ancestry	Candidate gene-based GWAS validation and functional study investigating common CYP17A1 variants linked to HTN	CYP17A1: rs138009835, rs2150927, rs2486758	Three SNPs (rs138009835, rs2150927, and rs2486758) had significant functional effects on CYP17A1 transcription. rs138009835 is in strong linkage disequilibrium with rs1004467, the top GWAS variant for BP. Carriers of the major G allele of rs138009835 had higher aldosterone excretion, suggesting a mechanistic link. LD Block 1 (rs2150927, rs2486758) linked with reduced 17α-hydroxylase efficiency; LD Block 2 (rs138009835) linked with elevated aldosterone synthesis	Adrenal steroidogenesis/RAAS (aldosterone/angiotensin)	Common promoter polymorphisms at the CYP17A1 locus, particularly rs138009835, modulate gene transcription and steroid phenotype, providing a functional explanation for HTN GWAS signals and highlighting the role of adrenal steroid biosynthesis in BP regulation
Kato et al. (2015) [61]	Total sample: up to 320,251 individuals. Ancestries: East Asian (31,516), European (35,352), South Asian (33,126) in discovery; replication in 133,052 more. Combined ancestry meta-analysis across all cohorts.	Trans-ancestry GWAS with replication and integrated methylation analysis	rs740406 (AMH), rs12046278 (IGFBP3), rs13359291 (KCNK3), rs11191548 (PDE3A), rs11065979 (PRDM6), rs653178 (ATXN2-SH2B3 region), rs1327235 (ARHGAP24), rs12686420 (OSR1), rs9801750 (SLC22A7), rs8049439 (TBX2)	Genetic risk scores from all BP loci predicted increased left ventricular mass, elevated NT-proBNP, and higher cardiovascular and all-cause mortality	Immune/inflammatory signaling (e.g., SH2B3)	This trans-ancestry GWAS revealed 12 new BP loci, highlighting epigenetic regulation, particularly DNA methylation, as a mechanistic link between genetic variation and HTN risk across diverse populations
Lu et al. (2015) [62]	Discovery cohort: 11,816 Han Chinese from six GWAS studies (e.g., InterASIA, GenSalt, NHAPC). Replication cohorts: 69,146 individuals across three independent studies. Total: 80,962 Chinese participants	GWAS with multi-stage replication in Chinese Han populations	rs9810888 (CACNA1D), rs2021783 (CYP21A2), rs11067763 (MED13L), rs820430 (SLC4A7), rs9266359 (HLA-B)	Novel genetic variants associated with BP and HTN	Vascular calcium handling/smooth-muscle contractility, renal tubular Na/Cl/HCO3⁻ transport (TAL/DT)	This large Chinese GWAS identified three novel loci (CACNA1D, CYP21A2, MED13L) and a Chinese-specific variant near SLC4A7, advancing understanding of shared and population-specific genetic mechanisms underlying BP regulation
Zhu et al. (2015) [63]	19 African ancestry cohorts (n ≈ 29,378), analyzing SBP, DBP, and HTN summary statistics from COGENT BP GWAS	Multi-trait meta-analysis of GWAS summary statistics	Four genome-wide significant loci: CHIC2 (Chr 4), HOXA-EVX1 (Chr 7), IGFBP1/IGFBP3 (Chr 7), CDH17 (Chr 8). Six additional loci showed suggestive evidence: CACNA1D, CYB5R2, HSF2/PKIB, PLXNC1, WNT3	CHIC2: opposite effects on SBP and DBP. HOXA-EVX1: consistent elevation of SBP and DBP. IGFBP1/IGFBP3: heterogeneous effects. CDH17: opposite directional effects on SBP and DBP	Vascular calcium handling/smooth-muscle contractility, cerebrovascular/endothelial remodeling	These four loci act through distinct but converging pathways: vascular remodeling (CHIC2, HOXA-EVX1), endocrine-metabolic regulation (IGFBP1/3), renal sodium and fluid balance (CDH17)
Frau et al. (2014) [64]	722 screened; 494 genotyped; 372 analyzed. White Caucasian adults (Italy and Sardinia), 18-59 years, mild-to-moderate essential HTN. Treatment: 50 mg/day losartan (ARB) for 4 weeks	Pharmacogenomic GWAS on 372 essential hypertensive patients treated with losartan for 4 weeks after an 8-week run-in diet-controlled phase; replication in two independent cohorts (GERA2, GENRES)	rs10752271 (CAMK1D)	rs10752271 (CAMK1D) as a novel genetic determinant of BP response to losartan	Unspecified/association not clearly mapped	rs10752271 in CAMK1D is a potential pharmacogenomic biomarker for personalized antihypertensive therapy
Chiang et al. (2014) [65]	992 matched Han Chinese case-control pairs. HTN onset before age 51; controls age- and sex-matched normotensive individuals	Three-stage GWAS on YOH in Han Chinese	C1orf135: rs213621-rs1257163 region; LARS: rs4913057-rs248779 region (Chr 5q32); GSN: rs12340264-rs306777 region (Chr 9q34); ACTN4: rs732135-rs973009 region (Chr 19q13, intron 1)	ACTN4, LARS, and GSN loci remained significant across multiple stages and partially replicated in HKHS and WTCCCHS cohorts. ACTN4 and LARS associated with SBP and HTN in replication samples	Unspecified/association not clearly mapped	Multi-stage integrative GWAS identified ACTN4, LARS, GSN, and C1orf135 as significant loci for young-onset HTN in Han Chinese, emphasizing ACTN4 as a robust cross-population candidate and demonstrating the value of combining multilocus association with gene expression analysis
Qi et al. (2014) [66]	1,765 participants (1,009 hypertensive, 756 normotensive controls)	Replication case-control GWAS	rs17030613, rs16849225, rs6825911, rs1173766, rs11066280, rs35444, rs880315, rs16998073, rs11191548, rs17249754	Three SNPs showed replicable associations: rs35444 near FES/FURIN (protective allele), rs11191548 near CYP17A1 (risk allele), rs17249754 near ATP2B1 (risk allele)	Vascular calcium handling/smooth-muscle contractility, adrenal steroidogenesis/RAAS (aldosterone/angiotensin)	Confirms the association of CYP17A1, ATP2B1, and FURIN/FES loci with HTN in Han Chinese, reinforcing their role in BP regulation and validating cross-ethnic consistency for several East Asian BP loci
He et al. (2013) [67]	1,881 participants in the GenSalt study and 698 in the independent replication cohort, Han Chinese	GWAS and meta-analysis; sequential dietary sodium/potassium interventions and cold pressor test	rs1330225 (PRMT6), rs10930597 (CDCA7), rs8002688 (PIBF1), rs16890334 (IRAK1BP1), rs11887188 (ARL4C), rs2030114 (SALL1), rs7577262 (TRPM8), rs17135875 (FBXL13)	rs1330225 (PRMT6) linked to high DBP/MAP during low-sodium; rs10930597 (CDCA7) and rs8002688 (PIBF1) linked to high MAP during low-sodium/potassium; rs16890334 (IRAK1BP1) linked to high SBP during high-sodium/potassium; rs11887188 (ARL4C) and rs2030114 (SALL1) linked to DBP during potassium; rs7577262 (TRPM8) linked to SBP response to cold pressor test; rs17135875 (FBXL13) linked to MAP response to cold pressor test. Dose-response observed between risk alleles and incident HTN over 7.5 years	Immune/inflammatory signaling (e.g., SH2B3)	PRMT6, CDCA7, IRAK1BP1, and SALL1 may mediate sodium/potassium regulation; TRPM8 encodes a cold-activated cation channel influencing sympathetic activation; PIBF1 and ARL4C may influence BP via immune and metabolic pathways
Xi et al. (2013) [68]	619 hypertensive vs. 2,458 normotensive participants (6-18 years) from the BCAMS study	Population-based case-control study examining obesity’s modification of genetic associations with BP/HTN	rs17249754 (ATP2B1), rs1378942 (CSK), rs1004467 (CYP17A1), rs3754777 (STK39), rs16998073 (FGF5), rs1801133 (MTHFR)	Normal-weight children: no significant associations. Overweight: STK39 rs3754777 associated with higher DBP; GRS correlated with higher SBP. Obese: SNP associations between SBP and ATP2B1 rs17249754, CSK rs1378942, CYP17A1 rs1004467; HTN associated with ATP2B1 rs17249754, CSK rs1378942, CYP17A1 rs1004467, MTHFR rs1801133 (OR = 1.22, P = 0.03)	Vascular calcium handling/smooth-muscle contractility, adrenal steroidogenesis/RAAS (aldosterone/angiotensin), endothelial/vascular signaling (NO-cGMP, Src/CSK), growth-factor/ubiquitin signaling (FGF5, HECTD4)	Obesity amplifies the genetic risk of HTN in Chinese children, revealing significant gene-environment interactions for ATP2B1, CSK, CYP17A1, STK39, and MTHFR
Miyaki et al. (2012) [69]	735 Japanese men from two occupational cohorts (Kanagawa and Kyoto)	Replication association study assessing 16 polymorphisms from 12 HTN-susceptibility genes, plus combined genetic effect analysis	rs4680, rs4633 (COMT); rs17249754 (ATP2B1); rs11191548 (CYP17A1); rs1378942 (CSK)	12 variants analyzed jointly → significant cumulative association with HTN prevalence and BP levels	Vascular calcium handling/smooth-muscle contractility, adrenal steroidogenesis/RAAS (aldosterone/angiotensin), endothelial/vascular signaling (NO-cGMP, Src/CSK)	Confirms individual and combined effects of multiple GWAS-identified variants (COMT, ATP2B1, CYP17A1, CSK, CYP11B2, PTGIS) on HTN and BP variation, highlighting the polygenic and additive nature of HTN susceptibility
Padmanabhan et al. (2009) [70]	Discovery cohorts: Framingham Heart Study, ARIC, CHS, Rotterdam, etc. (~29,136 participants). Replication: 34,433 individuals. Total >63,000 participants	Large-scale GWAS and meta-analysis investigating BP and HTN in European ancestry	rs13333226 (UMOD), rs11191548 (CYP17A1/NT5C2), rs16998073 (FGF5), rs2681472 (ATP2B1), rs1327235 (CSK/ULK3), rs381815 (ZNP510/LOC100129779), rs6015450 (PLEKHA7), rs2398162 (CACNB2)	Each risk allele increased SBP by ~0.8-1.2 mmHg, DBP by ~0.5-0.8 mmHg; weighted GRS combining 8 loci raised mean SBP by 4.6 mmHg and doubled HTN risk (OR ≈ 1.9)	Vascular calcium handling/smooth-muscle contractility, renal tubular Na/Cl/HCO3⁻ transport (TAL/DT), adrenal steroidogenesis/RAAS (aldosterone/angiotensin), endothelial/vascular signaling (NO-cGMP, Src/CSK), growth-factor/ubiquitin signaling (FGF5, HECTD4)	Identified eight novel loci (UMOD, ATP2B1, CYP17A1, CSK, FGF5, PLEKHA7, CACNB2, ZNP510) significantly associated with BP and HTN, revealing new pathways in renal sodium handling, calcium signaling, and vascular tone regulation
Hong et al. (2009) [71]	7,551 unrelated individuals (quantitative) and 8,512 (case-control)	Replication GWAS assessing six SNPs identified in WTCCC for essential HTN in a Korean population	rs6997709, rs7961152 (BCAT1), rs2820037, rs2398162, rs11110912, rs1937506	rs6997709 (protective), rs7961152 (risk), rs2820037 (protective)	Unspecified/association not clearly mapped	Korean replication of WTCCC GWAS found two SNPs (rs6997709, rs7961152) significantly associated with BP traits, with rs7961152 specifically linked to HTN; highlights BCAT1 as a candidate gene and ethnic differences in HTN genetics
Kato et al. (2011) [72]	Discovery: 19,608 East Asians (Japan, Korea, China, Taiwan); Replication: 10,445 participants. Total: 30,053	Large-scale GWAS and meta-analysis to identify BP and HTN loci in East Asians	rs2681492 (ATP2B1), rs11066280 (HECTD4), rs16998073 (FGF5), rs11191548 (CYP17A1/NT5C2), rs3184504 (SH2B3), rs13333226 (UMOD)	rs2681492 (ATP2B1) was the strongest novel locus; each risk allele increased SBP by 0.9-1.5 mmHg, DBP by 0.5-0.9 mmHg; the top decile of risk alleles had 1.6-fold higher HTN risk	ADRA1A-mediated adrenergic vasoconstriction pathway	Identified six loci robustly associated with BP and HTN in East Asians, with ATP2B1 and HECTD4 showing strong East Asian-specific effects, highlighting shared and population-specific genetic components of HTN
Takeuchi et al. (2010) [73]	25,826 Japanese participants (49.7% female, 18-97 years)	Multi-stage GWAS and replication analysis	rs880315 (CASZ1), rs17367504 (MTHFR), rs155524 (ITGA9), rs16998073 (FGF5), rs12413409 (FGF5), rs12413409 (CYP17A1-CNNM2), rs2681472 (ATP2B1), rs1378942 (CSK-ULK3)	Seven SNPs showed significant and reproducible associations with SBP, DBP, and/or HTN	Vascular calcium handling/smooth-muscle contractility, renal tubular Na/Cl/HCO3⁻ transport (TAL/DT), adrenal steroidogenesis/RAAS (aldosterone/angiotensin), immune/inflammatory signaling (e.g., SH2B3), growth-factor/ubiquitin signaling (FGF5, HECTD4)	Results support a multifactorial genetic basis for HTN, driven by coordinated influences on vascular smooth-muscle contractility, renal salt handling, and RAAS signaling
Yang et al. (2009) [74]	Han Chinese adults from Taiwan; Stage 1: 175 YOH cases, 175 controls; Stage 2: 1,008 cases, 1,008 controls	Two-stage GWAS followed by CMAS	rs9308945, rs6711736, rs6729869, rs10495809 (near LOC344371 and RASGRP3); interaction between rs1886985 (IMPG1) and rs6129969 (intergenic, chr20)	Identified gene variants and interactions showed a strong association with young-onset HTN	Unspecified/association not clearly mapped	Suggests young-onset HTN has a polygenic and interactive genetic architecture, involving additive and synergistic SNP effects; highlights RASGRP3 and IMPG1 as novel HTN-associated genes in Han Chinese
Ehret et al. (2008) [75]	11,433 genotyped individuals from 12,593 across GenNet, GENOA, HyperGEN; ethnic groups: AA, EA, HA	Replication study of WTCCC HTN GWAS using Family Blood Pressure Program data	rs1937506 (chr13q21)	EA: G allele associated with -24.9 mmHg SBP, -8.7 mmHg DBP (protective). HA: G allele associated with +27.7 mmHg SBP (opposite direction)	Unspecified/association not clearly mapped	Only one SNP showed suggestive association with SBP and DBP, but effects were opposite across ethnic groups, underscoring ethnic specificity of HTN genetics
Levy et al. (2007) [76]	1,327 participants (mean age 52 years; 54% women) from Framingham Heart Study Offspring Cohort	GWAS	rs10493340 (chr1), rs1963982 (chr8), rs10491334 (CAMK4), rs2121070 (C14orf118), rs6063312 (PREX1), rs770189 (MEF2C), rs1322512 (SYNE1), rs10507514 (TNFSF11)	No genome-wide significant associations; several loci showed suggestive signals for BP and arterial stiffness	Unspecified/association not clearly mapped	Early GWAS identified MEF2C, SYNE1, TNFSF11, and LOXL2 as potential contributors to HTN and arterial stiffness, highlighting the polygenic and structural complexity of BP regulation

Key Replicating Signals and Convergent Pathways

Multiple GWAS have repeatedly identified the same genes associated with blood pressure. On the vascular side, variants in the L-type calcium-channel genes CACNA1D/CACNB2 [30,34,38,43,55,56,63,70] and the plasma-membrane calcium pump ATP2B1 were consistently observed [58,66,68,72]. These proteins regulate calcium flux in vascular smooth-muscle cells, thereby determining contractile strength and arteriolar (myogenic) tone, which in turn influences blood pressure. Renal loci such as UMOD (uromodulin, expressed in the thick ascending limb) [70,72] and SLC4A7 (a bicarbonate transporter) affect tubular handling of sodium and bicarbonate [32,55,62]. By altering sodium and water reabsorption, they modify effective circulating volume and thus baseline blood pressure set points rather than short-term fluctuations. Endocrine loci CYP17A1 and CYP11B2 encode enzymes in adrenal steroidogenesis, including aldosterone; variation at these sites modulates the RAAS, with downstream effects on vascular tone and renal sodium reabsorption [36,58,60,66,68,70,72]. Immune signaling is represented by SH2B3, an adaptor in JAK/STAT-related pathways that links low-grade inflammation to vascular stiffness, endothelial dysfunction, and altered renal salt handling [32,53,55,58,61,72]. These associations have been observed across large European- and East Asian-led studies and remain robust after fine-mapping, which narrows signals to likely causal variants, and tissue-specific annotation, which demonstrates effects in relevant tissues such as the artery, kidney, and adrenal. The consistency across ancestries and analytic approaches strengthens the inference that these pathways are central to blood pressure regulation.

Ancestry, Transferability, and Fine-Mapping

Multi- or trans-ancestry meta-GWAS (pooled GWAS that combine data from multiple ancestral groups) improved the precision of genetic mapping by leveraging differences in LD (i.e., how often nearby variants are inherited together) [29,32-34,36,40,45,47]. When LD patterns differ across ancestries, a risk signal that spans many correlated variants in one group can be narrowed in another, which reduces the “credible set” (the smallest set of variants that is statistically likely to contain the causal one) [34,36,43,47,61,72]. In practice, this means that cross-ancestry analyses more precisely localized blood pressure loci while still pointing to the same underlying biology across populations [29,32,33,47]. East Asian datasets were especially informative for fine mapping (the statistical process of narrowing from broad regions to likely causal variants) [34,36,43,61,72]. Their LD structure sharpened signals at HECTD4, FGF5, ATP2B1, and within the UMOD region [34,36,43,61,72]. Many of these refined signals also “transferred” to European cohorts, i.e., they reproduced in European data, supporting their general validity and relevance beyond a single population [29,32-34,36,43,47,61,72]. These analyses also identified ancestry-enriched variants (alleles that are more frequent or better tagged in one ancestry and therefore easier to detect there) [31,34,47]. Importantly, the presence of such variants did not imply different biological mechanisms across ancestries. For most well-replicated (“canonical”) loci, effect size heterogeneity (small differences in the magnitude of genetic effects between groups) was modest [32,33,47]. This pattern argues for ancestry-aware modeling, such as estimating and calibrating effects using diverse datasets, rather than assuming ancestry-specific pathophysiology [30,51,63]. Overall, multi-/trans-ancestry meta-GWAS increased fine-mapping resolution, revealed some ancestry-enriched signals, and preserved a shared mechanistic core for blood pressure regulation (vascular calcium handling, renal salt transport, adrenal steroidogenesis, and immune signaling) [29-34,36,40,43,45,47,51,61,63,72]. This combination improves both biological interpretation and the portability of genetic findings across populations.

From Association to Mechanism

Across the studies listed in Table 2, GWAS signals were translated into mechanisms by layering four complementary approaches, such as eQTL colocalization, Mendelian randomization, regulatory (chromatin/3D) annotation, and targeted perturbation, and by reading their results against tissue context (artery, kidney, and adrenal) [31,33,44,49,65,66]. Taken together, these analyses converged on a coherent map in which vascular calcium handling, renal salt/bicarbonate transport, adrenal steroidogenesis, and immune signaling underpin blood pressure regulation.

Linking loci to effector genes via expression. eQTL colocalization repeatedly showed that the same variants associated with blood pressure are also associated with gene expression in disease-relevant tissues, thereby nominating proximate effector genes. Signals at the ATP2B1 locus colocalized with arterial expression, supporting a role for vascular smooth-muscle calcium efflux and arteriolar tone; signals at the UMOD locus colocalized with kidney expression in the thick ascending limb, implicating tubular sodium handling and volume control [31,33,49,65].

Establishing causality from metabolic traits to BP. Using Mendelian randomization, multiple studies tested whether adiposity, lipid levels, and glycemic traits causally raise blood pressure rather than merely correlate with it. The instruments supported directional effects (e.g., higher genetically predicted BMI or LDL linked to higher BP), explaining why cardiometabolic loci often overlap BP loci and how metabolic load can shift vascular and renal physiology toward higher pressure [31,33,66].

Placing variants in active regulatory DNA. By overlaying GWAS peaks with open chromatin, enhancer marks, transcription-factor motifs, and promoter-enhancer contacts, sentinel variants were located within active regulatory elements in artery, kidney, and (for RAAS) adrenal tissue. This showed that the implicated variants sit in DNA regions that actually regulate transcription, where the trait biology resides, tightening the link from variant to gene regulation and phenotype [44,49,65].

Functionally testing the mechanism. Targeted perturbation means experimentally changing the activity of a candidate gene to see if the cell behaves as the genetic and regulatory evidence would predict. In practice, investigators used tools such as CRISPR (to edit or knock out a gene), knockdown (to reduce its expression with RNA-based methods), or overexpression (to increase its expression) and then measured relevant cellular readouts, the immediate physiological outputs of the pathway in the correct cell type. For blood pressure loci, two illustrative examples emerged. First, perturbing ATP2B1 in vascular smooth-muscle cells altered intracellular calcium handling and contractility, consistent with the gene’s role as a plasma-membrane Ca²⁺ pump that helps set arteriolar tone. Second, manipulating UMOD in renal thick ascending limb models changed tubular sodium handling, aligning with uromodulin’s influence on salt reabsorption and volume status. Because these perturbation results point in the expected direction and are observed in the right tissues, they provide orthogonal, causal support, independent of statistical association or regulatory annotation, which strengthens the inference from GWAS locus to mechanism [42,49].

Integrated inference and therapeutic relevance. Because these methods are independent yet pointed to the same themes, such as (i) vascular Ca²⁺ handling (ATP2B1, calcium-channel loci), (ii) renal salt/bicarbonate transport (UMOD, SLC4A7), (iii) adrenal steroidogenesis/RAAS (CYP17A1, CYP11B2), and (iv) immune signaling (SH2B3), the combined evidence is stronger than any single line alone. Colocalization identifies which gene in which tissue; chromatin shows the variant sits in an active regulator there; MR shows metabolic exposures push BP causally; and perturbation demonstrates that changing the gene shifts the physiology as predicted. This multi-layer agreement supports the pharmacologic logic of calcium-channel blockade (vascular Ca²⁺ axis), mineralocorticoid antagonism (RAAS axis), and diuretic strategies (renal transport axis) as mechanism-aligned interventions [31,33,44,49,65,66].

Phenotype Refinement and Resistant Hypertension

Beyond “mean BP,” phenotype-focused analyses show that resistant hypertension is enriched for calcium-handling and steroidogenesis signals, paralleling clinical response to calcium-channel blockers and mineralocorticoid receptor antagonists; BP variability/ambulatory traits share core loci with mean BP but add modifiers of endothelial tone and reactivity, suggesting partially distinct architectures with prognostic implications [50,51]. These results argue for genetic sub-phenotyping when evaluating therapeutic choices and risk.

Pleiotropy and Cross-Trait Architecture

Pleiotropy, where a single genetic locus influences multiple traits, was systematically assessed in the Table 2 studies. These analyses showed that blood pressure loci frequently overlapped with adiposity, lipid, and glycemic traits, indicating shared biological pathways rather than isolated, trait-specific effects [32,46,53,58,63,66]. The cardiometabolic overlap was most evident at immune/vascular and renal-transport loci: SH2B3 is associated with BP and immune-metabolic phenotypes; calcium-handling genes (ATP2B1 and CACNA1D/CACNB2) link vascular reactivity to cardiometabolic profiles; and renal loci (UMOD and SLC4A7) connect tubular sodium/bicarbonate handling with both hypertension and metabolic traits [32,46,53,58]. Mendelian randomization further supported causal paths from adiposity, lipids, and glycemia to higher BP, reinforcing that these overlaps reflect shared mechanisms, not mere correlation [32,46,66]. Collectively, these findings support integrated cardiometabolic risk models and mechanism-aligned therapy, including avenues for drug repurposing [32,46,53,58,63,66].

Comparative Takeaways

Relative to earlier single-ancestry or candidate gene efforts, modern biobank-scale GWAS detect more loci with smaller effects and achieve better causal assignment through integrative functional data; compared with purely discovery-oriented scans, trans-ancestry frameworks yield finer maps and better polygenic portability; and versus non-integrative GWAS, studies that include eQTL/MR/perturbation offer clearer mechanistic bridges suitable for translation [31-33,47,58,76].

Clinical Significance

Collectively, the GWAS literature supports a polygenic architecture dominated by vascular Ca²⁺ dynamics, adrenal steroid output, renal tubular salt handling, and immune remodeling, with robust replication across ancestries. These results justify (i) ancestry-aware polygenic risk and subtyping to anticipate earlier onset and drug response; (ii) prioritization of calcium-channel and mineralocorticoid pathways in resistant phenotypes; and (iii) closer integration of renal handling and inflammatory tone into risk stratification, while ongoing multi-ancestry discovery and functional validation will further refine targetability and clinical utility [29,30,32-34,36,40,45,47,49-51].

WES and WGS insights into systemic hypertension

The integration of next-generation sequencing technologies, particularly WES and WGS, has significantly advanced the discovery of rare and population-specific variants influencing systemic hypertension. Unlike GWAS, which primarily detect common noncoding variants with small effect sizes, sequencing-based studies can uncover rare, functional, and ancestry-specific mutations with direct biological relevance. The following major studies collectively illustrate how sequencing approaches elucidate the molecular mechanisms and ethnic diversity underlying blood pressure regulation.

In an earlier exome-based study, Yu et al. analyzed 17,956 individuals of European and African ancestry from the CHARGE Consortium and NHLBI Exome Sequencing Project [77]. The study identified rare loss-of-function and missense variants in CLCN6, a chloride channel gene, which were associated with lower diastolic BP (−3.3 mmHg) and a 28% reduction in hypertension risk (OR = 0.72). Functional enrichment analyses localized most variants to domains regulating chloride ion transport, with parallels to CLCNKA and CLCNKB, genes known to cause Bartter and Gitelman syndromes, characterized by hypotension. This provided the first exome-wide evidence linking chloride channel dysfunction to BP reduction in the general population.

Armstrong et al. conducted a large multiethnic WGS study of 29,208 participants in the NHLBI Trans-Omics for Precision Medicine (TOPMed) program to explore the genetic basis of apparent treatment-resistant hypertension [78]. The authors identified both common and rare variants in KCNK3, SCN10A, NKAIN2, and ANGPT4-RSPO4, implicating potassium and sodium ion transport, endothelial signaling, and WNT pathways in resistance to antihypertensive therapy. In population-specific analyses, SCN10A variants were prominent among Europeans, while SGCZ-TUSC3 variants were enriched in African Americans. Gene-based rare variant testing highlighted MYL4, PDCD4, and ERG, linking cardiac contractility and endothelial stress regulation to hypertension pathophysiology. These findings emphasize vascular ion transport and endothelial integrity as central to resistant hypertension.

Similarly, Kelly et al. analyzed over 760,000 individuals across multiple global cohorts using WGS to identify novel rare variants influencing systolic and diastolic BP [79]. The study revealed a rare intergenic variant near LOC100506274 with a large BP-lowering effect (≈33 mmHg reduction in systolic BP) and a common variant in the INSR locus, reinforcing the link between insulin signaling and vascular tone regulation. Additional rare-variant associations in genes such as GABRB3, KIF3B, and ERBB4 pointed toward neuronal, endothelial, and calcium-signaling mechanisms in BP control. Together, these studies demonstrate the capacity of WGS to capture noncoding regulatory and ancestry-specific variants that may underlie interindividual differences in blood pressure and treatment response.

Adding regional diversity, Alsamman et al. performed whole-genome sequencing on 200 Qatari individuals (100 hypertensive and 100 normotensive), representing one of the first large-scale genetic investigations of essential hypertension in the Middle East [80]. The study identified novel loci, such as GMPS-SETP14, ISCA1P6-AC012451.1, and RBM47, and highlighted SPATS2L, ULK4, and FHIT as multi-SNP genes strongly associated with hypertension. Pathway analysis emphasized ion-gated channel activity, cardiac conduction, and renin-angiotensin signaling as major regulatory mechanisms. Cross-validation with over 10,000 published studies confirmed overlaps with known BP-related genes such as SCN5A, SCN10A, RYR2, and EPAS1. Notably, 194 genes were unique to the Qatari cohort, underscoring population-specific genetic signatures that could guide precision medicine in Arab populations.

Collectively, these findings underscore that hypertension is a polygenic, multi-pathway disorder influenced by both rare high-impact variants and common regulatory alleles. The inclusion of diverse populations, such as the Qatari cohort, has further revealed unique regional genetic profiles while reaffirming globally conserved biological mechanisms. Sequencing-based evidence thus enhances the foundation for precision hypertension management, enabling gene-based risk stratification and therapeutic targeting of molecular pathways like KCNK3, ENPEP, and CLCN6.

## Conclusions

Hypertension genetics shows a coherent, multi-pathway architecture in which modest-effect common variants (GWAS) and select rare, functional alleles (sequencing) converge on (i) vascular Ca²⁺ flux and smooth-muscle contractility; (ii) renal tubular salt/bicarbonate transport; (iii) adrenal steroidogenesis/RAAS; and (iv) immune/inflammatory signaling. These axes explain cross-trait pleiotropy with metabolic traits, ancestry-modulated effect sizes, and the enrichment of resistant-hypertension signals in calcium/steroidogenesis loci. The compiled evidence argues for ancestry-aware polygenic tools, genetic sub-phenotyping (especially for resistant phenotypes), and mechanism-aligned therapy as practical routes to precision hypertension care.
